# Systematic literature review and meta-analysis on the epidemiology of methylmalonic acidemia (MMA) with a focus on MMA caused by methylmalonyl-CoA mutase (mut) deficiency

**DOI:** 10.1186/s13023-019-1063-z

**Published:** 2019-04-25

**Authors:** Tímea Almási, Lin T. Guey, Christine Lukacs, Kata Csetneki, Zoltán Vokó, Tamás Zelei

**Affiliations:** 1 Syreon Research Institute, Mexikói str. 65/A, Budapest, H-1142 Hungary; 2 Moderna, Inc., Cambridge, MA USA; 3 Department of Health Policy & Health Economics, Eötvös Loránd University, Budapest, Hungary

**Keywords:** Inherited metabolic disorder, Methylmalonic acidemia/aciduria, Methylmalonyl-CoA mutase deficiency, Epidemiology, Meta-analysis, Newborn screening

## Abstract

Methylmalonic acidemia/aciduria (MMA) is a genetically heterogeneous group of inherited metabolic disorders biochemically characterized by the accumulation of methylmalonic acid. Isolated MMA is primarily caused by the deficiency of methylmalonyl-CoA mutase (MMA mut; EC 5.4.99.2). A systematic literature review and a meta-analysis were undertaken to assess and compile published epidemiological data on MMA with a focus on the MMA mut subtype (OMIM #251000). Of the 1114 identified records, 227 papers were assessed for eligibility in full text, 48 articles reported on disease epidemiology, and 39 articles were included into the quantitative synthesis. Implementation of newborn screening in various countries has allowed for the estimation of birth prevalence of MMA and its isolated form. Meta-analysis pooled point estimates of MMA (all types) detection rates were 0.79, 1.12, 1.22 and 6.04 per 100,000 newborns in Asia-Pacific, Europe, North America and the Middle East and North Africa (MENA) regions, respectively. The detection rate of isolated MMA was < 1 per 100,000 newborns in all regions with the exception of MENA where it approached 6 per 100,000 newborns. Few studies published data on the epidemiology of MMA mut, therefore no meta-analysis could have been performed on this subtype. Most of the identified papers reported birth prevalence estimates below 1 per 100,000 newborns for MMA mut. The systematic literature review clearly demonstrates that MMA and its subtypes are ultra-rare disorders.

**Electronic supplementary material:**

The online version of this article (10.1186/s13023-019-1063-z) contains supplementary material, which is available to authorized users.

## Background

Methylmalonic acidemia or aciduria (MMA) is a genetically heterogeneous group of disorders originating from impaired metabolism of certain amino acids (isoleucine, methionine, threonine, or valine), odd-chain fatty acids or cholesterol esters. MMA is biochemically characterized by the accumulation of methylmalonic acid in all body fluids and tissues [1]. Two main forms can be distinguished: isolated MMA and combined MMA. The isolated form may be caused by a complete or partial deficiency of the enzyme methylmalonyl-coenzyme A (CoA) mutase (mut; mut^0^ enzymatic subtype or mut^−^ enzymatic subtype, respectively) (EC 5.4.99.2) (Online Mendelian Inheritance in Man (OMIM) number *609058), a defect in the transport or synthesis of its cofactor, adenosyl-cobalamin (cblA, cblB, cblD-MMA, cblH), or by a deficiency of the enzyme methylmalonyl-CoA epimerase [2]. Combined MMA presents with homocystinuria/ homocystinemia (cblC, cblD-MMA/HC, cblF, cblJ) and also with malonic acidemia/aciduria (CMAMMA type) [3]. The majority of MMA patients present with clinical signs and symptoms within the first few days or weeks of life [4, 5] and the overall prognosis is generally poor, with the occurrence of intermittent life-threatening acute metabolic decompensations precipitated by catabolic factors and significant long-term sequelae including neurologic and renal impairment [6, 7].

The inclusion of MMA in newborn screening panels has allowed for early diagnosis. The impact of newborn screening on the long-term outcomes of MMA remains to be fully elucidated [7], however according to the European registry and network for Intoxication type Metabolic Disorders (E-IMD), newborn screening is effective in reducing the time to diagnosis for late-onset patients and reduces the likelihood of movement disorders in MMA patients who are responsive to cobalamin supplementation [8].

Although newborn screening studies have been published in different regions, a systematic review of the epidemiology literature in MMA has not been performed to date. Thus, a systematic literature review (SLR) followed by a meta-analysis was undertaken to compile and assess published epidemiological data on methylmalonic acidemia (MMA) with a focus on the isolated form caused by methylmalonyl-CoA mutase deficiency (MMA mut).

## Methods

### Systematic literature review

**Fig. 1:**
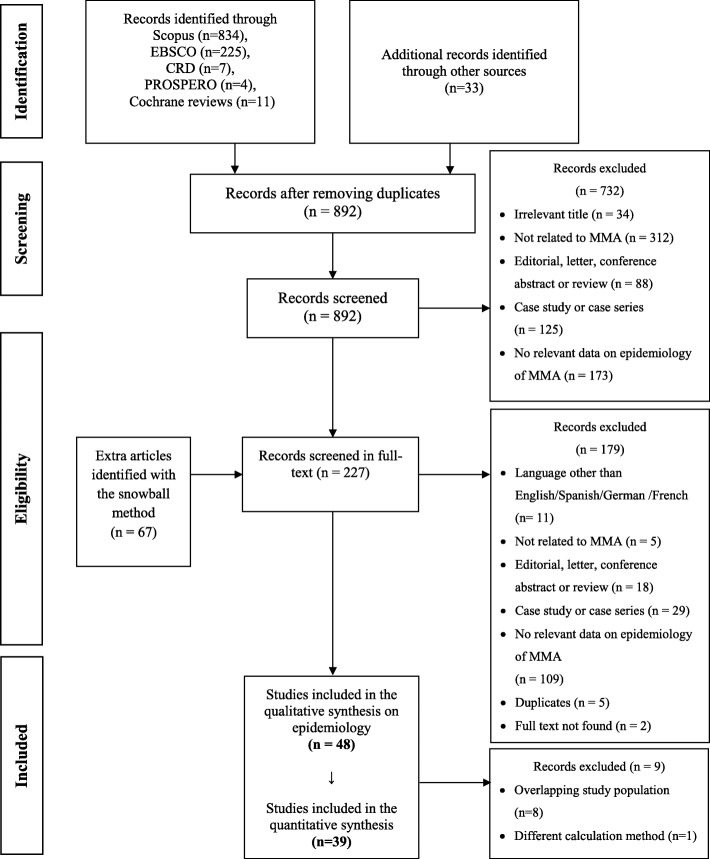
Flow of information diagram

The literature search was performed covering Medline, Embase, Cochrane Database of Systematic Reviews, Centre for Reviews and Dissemination (CRD) Database, Academic Search Complete, Cumulative Index to Nursing and Allied Health Literature (CINAHL) and PROSPERO databases. Websites of rare disease organizations were also searched for eligible studies. The search strategies used in scientific databases with the date of the search and number of hits are summarized in Table S1 [see Additional file 1]. The exclusion criteria of the title/abstract screening and full-text reviews are summarized in Fig. 1 and are detailed in Table S2 [see Additional file 1]. A snowball method was also used to identify further relevant studies among the references of full text papers.

Data extraction was performed by two independent researchers, and conflicts were resolved by discussion until a consensus was reached. Published data were considered relevant for the disease epidemiology if the reference population – from which the cases were identified – was representative of the general population of the investigated country or region. Reports on national screening programs with ~ 100% population coverage and analyses of national statistics were considered to provide the most accurate data on disease epidemiology. Reports on screening programs not covering ~ 100% of the population were considered to be eligible if a relatively large, random sample was used or the screening program had a multicenter design. Studies reporting on selected patient populations (e.g. patients with clinical suspicion of inborn error of metabolism) were excluded. Risk of bias was evaluated on study-level, using the tool developed by Hoy et al. [9] designed to assess methodology and risk of bias in prevalence studies [9]. The checklist consists of 10 close-ended questions assessing the overall risk of study bias both on the level of internal and external validity. Risk of bias was assessed for all studies. A summary score of 0–3 was indicated as low risk, 4–6 as moderate risk and 7–10 as high risk.

### Meta-analysis

Studies with moderate or low risk of bias as determined by the Hoy tool were eligible for the quantitative synthesis [9]. Overlap among the study populations across multiple studies were rigorously investigated by reviewing countries/regions, study periods, data sources and study cohorts. Overlapping populations were excluded and the publication with the more complete dataset was included in the meta-analysis.

Random effects meta-analysis was performed including all identified studies presenting disease occurrence data, regardless of the calculation method used (i.e. birth prevalence, lifetime risk and cumulative incidence). Heterogeneity between the individual study estimates was determined by the chi-square test and the I-square (I^2^) statistics. The Metaprop module for STATA was used to perform all meta-analyses in STATA SE 15.0. This routine provides procedures for pooling proportions in a meta-analysis of multiple studies. The confidence intervals of the individual study estimates are based on the exact binomial (Clopper-Pearson) procedure [10]. Confidence intervals for the pooled estimate were calculated after Freeman-Tukey double arcsine transformation.

The meta-analysis was carried out for the following regions: North America, Europe, Asia-Pacific, Middle East and North Africa. Time trends were analyzed by performing a subgroup analysis covering three different time periods: − 1980, 1981–2000, 2001-present. In order to decrease the heterogeneity of epidemiological measures, a sensitivity analysis was undertaken by excluding studies not presenting birth prevalence data.

## Results

A total of 1114 records were identified by the literature searches. After duplicates were removed, 892 records were screened by their titles and abstracts from which 160 articles qualified for a full-text review. The snowball method identified 67 extra articles which were mainly newborn screening reports that did not mention MMA-related terms in their title, abstract or keywords, and therefore were not identified by the search strategy. In total, 227 articles were assessed for eligibility in full text and 48 were considered eligible for the qualitative synthesis (Fig. 1). Among the 48 articles there were 8 overlapping studies [11–18] and one article used a different calculation method than the remaining articles [19]; these were excluded from the quantitative analysis.

The literature review provided data from 25 countries from 4 different geographic regions. The majority of the publications originated in developed countries.

A variety of epidemiological terms were used across the studies to report the proportion of newborns who were or would be affected by MMA. Due to this heterogeneity the reported measures were categorized based on their calculation methods into scientifically acceptable epidemiological terms (Table S3) [see Additional file 1]. The vast majority of articles reported on newborn screening programs, providing estimates on birth prevalence, defined as the number of affected newborns divided by the total screened population. Three articles followed a specific birth cohort over time and counted the number of diagnoses over the follow-up period, providing estimates on the cumulative incidence in the birth cohort [11, 20, 21]. In six cases, authors estimated the lifetime risk at birth – a special case of cumulative incidence in which the period of time studied is the entire remaining lifetime – by dividing the newly diagnosed cases in an observational period by the number of all live births during the same time period [22–28]. Although the calculation methods differ, the difference in the results is small if it is assumed that MMA appears early in life, the disease occurrence is more or less constant, the size of birth cohorts and the diagnostic methods did not change significantly over time and all patients who have the underlying mutation will present with clinical symptoms over their lifetime. Based on these assumptions, we use the term “detection rate” for the three above-mentioned measures throughout the paper. One study reported the proportion of affected patients divided by the total population at a certain date providing point prevalence data for the disease [19]. Point-prevalence is not comparable to the three above-mentioned calculation methods, therefore it was omitted from the quantitative synthesis.

### Epidemiology data on MMA (all types)

**Fig. 2:**
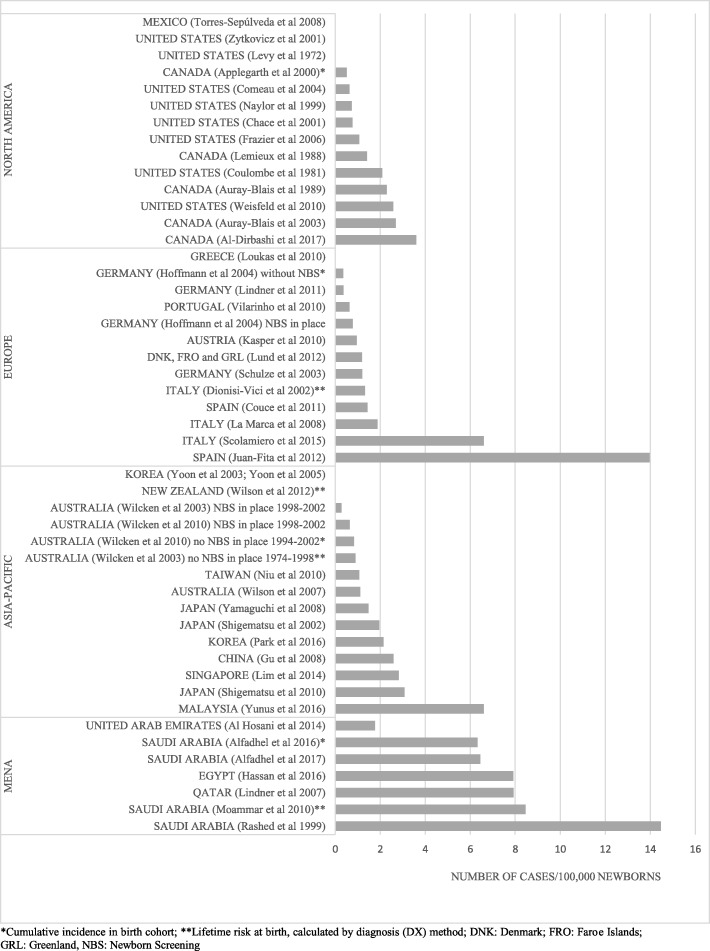
Estimates on birth prevalence of methylmalonic acidemia. *Cumulative incidence in birth cohort; **Lifetime risk at birth, calculated by diagnosis (DX) method; DNK: Denmark; FRO: Faroe Islands; GRL: Greenland, NBS: Newborn Screening

**Fig. 3:**
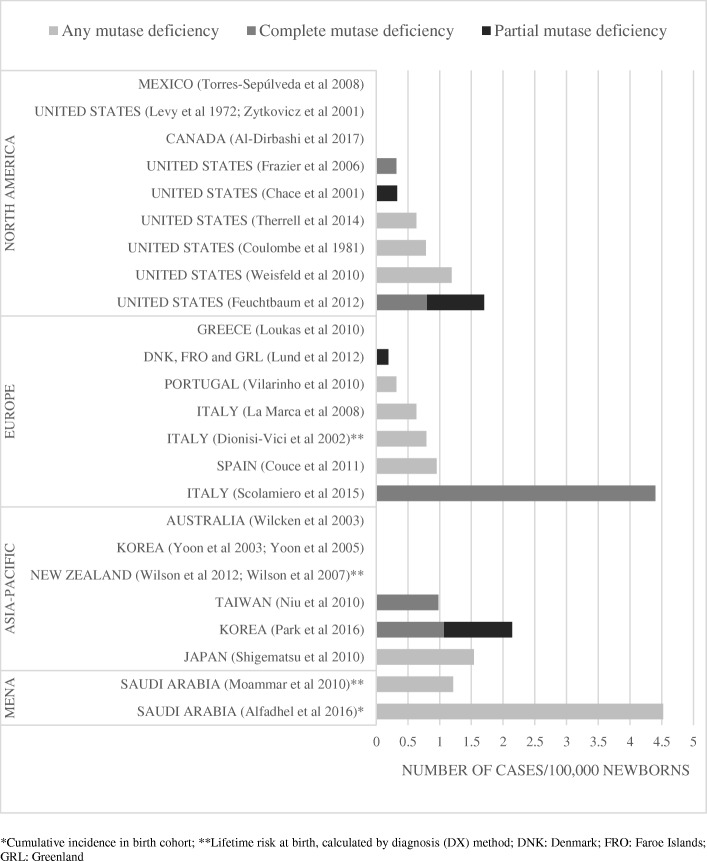
Estimates on birth prevalence of methylmalonic acidemias caused by mutase deficiency. *Cumulative incidence in birth cohort; **Lifetime risk at birth, calculated by diagnosis (DX) method; DNK: Denmark; FRO: Faroe Islands; GRL: Greenland

The pooled point estimates of the detection rates were 0.79 (CI: 0.44–1.21), 1.12 (CI: 0.50–1.91), 1.22 (CI: 0.61–2.01) and 6.04 (CI: 4.02–8.41) per 100,000 newborns in Asia-Pacific, Europe, North America and MENA, respectively. Regional and country-specific detection rates are provided in Fig. 2 [11–18, 20, 21, 23–59] and Fig. 3 [11, 16–18, 23, 25–30, 32, 39–45, 47, 50–54, 60, 61]. Results of the meta-analyses along with the conducted sensitivity analyses can be found in Table 1.

**Table 1:** Base case and sensitivity analysis by geographic area (SA includes only studies with birth prevalence measure) (detection rate per 100,000 newborns)

Base case							Sensitivity analysis (birth prevalence)					
	MMA			Isolated MMA			MMA			Isolated MMA		
Region	point estimate (95% CI)	Number of studies	I^2^	point estimate (95% CI)	Number of studies	I^2^	point estimate (95% CI)	Number of studies	I^2^	point estimate (95% CI)	Number of studies	I^2^
North America	1.22 (0.61–2.01)	10	79.79%	0.38 (0.09–0.79)	8	53.23%	1.36 (0.71–2.20)	9	76.46%	0.42 (0.09–0.91)	7	53.07%
Europe	1.12 (0.50–1.91)	11	79.22%	0.60 (0.16–1.23)	6	2.17%	1.55 (0.51–3.02)	9	79.95%	0.60 (0.16–1.23)	6	2.17%
Asia-Pacific	0.79 (0.44–1.21)	12 (15 cohorts)	45.46%	0.51 (0.32–0.74)	9 (12 cohorts)	0.00%	1.00 (0.46–1.69)	11	44.76%	0.57 (0.27–0.96)	8	0.00%
MENA	6.04 (4.02–8.41)	6	15.78%	5.69 (2.54–9.87)	2	NA	5.60 (2.94–8.94)	5	21.45%	NA	NA	NA

In North America the detection rate of MMA was the lowest in Massachusetts (< 0.45/100,000) and the highest in Ontario (3.60/100,000) [29, 30]. A minor increasing tendency was observed when analyzing different time periods in North America; the pooled point estimate was 0.96 (CI: 0.28–1.97) in the period of “-1980” while it was 1.53 (CI: 0.67–2.68) per 100,000 newborns in the period of “2001-present”.

Publications from Europe show a heterogeneous picture with two publications reporting relatively high figures from the Southern region. Juan-Fita et al. (2012) identified 10 MMA cases in Murcia, Spain during a pilot screening program covering 71,595 newborns that resulted in a detection rate of 13.97 per 100,000 newborns [31]. Authors explained this high number by the relatively high rate of immigration from North Africa and South America. Scolamiero et al. (2015) reported on a pilot screening program from Campania, Italy, in which 45,466 newborns were screened. They reported 3 identified MMA cases that resulted in a detection rate of 6.60 per 100,000 newborns [32]. European data were not available before 1980 and only a minor increase was observed between the period of “1981–2000” and “2001-present” (1.18 (CI: 0.95–1.43) vs. 1.55 (CI: 0.51–3.02) per 100,000 newborns).

In the Asia-Pacific region the detection rate of MMA was the lowest in New Zealand (< 0.54/100,000) and the highest in Malaysia (6.61/100,000) [27, 33]. The latter estimate was based on a pilot screening program including 11 public hospitals across the country which screened 30,247 newborns; thus the representativeness of this study is uncertain. No time trend was observed.

In the MENA region, a tendency towards increased detection rates was observed, with estimations ranging from 1.77 in the United Arab Emirates to 6.45–14.48 in Saudi Arabia per 100,000 newborns [11, 25, 34–38]. Time trends showed decreasing tendencies over the years; while the pooled point estimate was 8.66 per 100,000 newborns (CI: 4.79–13.54) in the period of 1981–2000, it decreased to 5.21 (CI: 3.67–6.97) per 100,000 newborns in the period of “2001-present”. However, these subgroup analyses included only a few (2 and 4 respective) studies. The only paper reporting point prevalence data was from Oman, where authors reported on 8 MMA patients, resulting in a point prevalence of 0.29 per 100,000 inhabitants [19].

### Epidemiology data published on isolated MMA

The pooled point estimates were 0.38 (CI: 0.09–0.79), 0.51 (CI: 0.32–0.74), 0.60 (CI: 0.16–1.23) and 5.69 (CI: 2.54–9.87) per 100,000 newborns in North America, Asia-Pacific, Europe and MENA, respectively.

In North America the lowest detection rate was published from Massachusetts by Levy et al. [29], where no cases were identified out of 222,302 infants [29]. The highest estimate was 1.59/100,000 newborns in New York State where 16 isolated MMA cases were detected among the 1,006,325 screened newborns [39]. No clear tendency was indicated by the time-specific subgroup analysis.

In Europe the highest estimate originated from Campania, Italy (4.40/100,000 newborns) and the lowest value was reported from Athens, Greece where no MMA cases were identified among the 45,000 screened newborns [32, 40]. As all identified articles reporting on the epidemiology of isolated MMA were published after 2001, no time-specific subgroup analysis was conducted.

In Asia-Pacific, the lowest estimate was < 0.28/100,000 newborns in New-Zealand [27], while the highest estimate was identified in South Korea (2.15/100,000 newborns) [41]. Time-specific subgroup analysis showed a marginal decrease between the period of “1981–2001” and “2001-present”.

In the MENA region, the detection rates were higher, ranging from 3.97 to 6.65 per 100,000 newborns [11, 25, 37]. No time-specific subgroup analysis could be conducted as both studies included into the meta-analysis were published after 2007.

### Epidemiology data published on MMA caused by mutase deficiency (MMA mut)

The literature reporting on the epidemiology of MMA caused by mutase deficiency was scarce and the data was heterogeneous. Therefore, no quantitative meta-analysis could be performed in the case of this subtype.

In the United States, Therrell et al. [60] reported aggregate statistics of newborn screening programs which covered 25,219,021 newborns in the period of 2001–2010 [60]. The authors detected 158 MMA mut cases, a detection rate of 0.63 per 100,000 newborns. Three other articles reported on screening programs covering > 900,000 newborns in North America where the detection rates of mut deficiency ranged between 0.32 and 1.19 per 100,000 newborns [39, 42, 43]. Regional and country-specific detection rates are provided in Fig. 3.

In Europe the detection rate varied between < 2.22/100,000 (Greece) and 4.4/100,000 (Italy) [32, 40]. The largest investigated population from the region indicated a detection rate of 0.79/100,000 newborns [23].

In the Asia-Pacific region, the estimate derived from the largest sample size (> 1,000,000 newborns) indicated a detection rate of 0.98 per 100,000 newborns in Taiwan [44].

Data from the MENA region indicated a detection rate between 1.21 and 4.52 per 100,000 newborns [11, 25].

Few studies were identified that reported data separately on the epidemiology of MMA due to partial (mut^−^) or complete (mut^0^) mutase deficiency. Estimations based on reference populations of > 900,000 newborns provided the following rates for MMA mut^−^; < 0.08/100,000 (Taiwan), < 0.11/100,000 (North Carolina), 0.33 (United States) and 0.9 per 100,000 newborns (California) [42–44, 61]. The most reliable estimates of MMA mut^0^ in reference populations of > 900,000 newborns are 0.32/100,000 in North Carolina, 0.8/100,000 in California and 0.98/100,000 in Taiwan [43, 44, 61].

## Discussion

Based on the meta-analyses, the detection rate of MMA and isolated MMA was below 2 cases per 100,000 newborns in North America, Europe and Asia-Pacific regions. In the region of MENA, the meta-analysis results indicated higher disease frequencies for MMA and its isolated form. These results are in line with the recent findings of Chapman et al. (2018) who reported a detection rate of 5.05, 1.44 and 0.18 per 100,000 newborns of isolated MMA in Kuwait, United States and South-West Germany, respectively [62].

The higher disease frequency in the MENA region is most likely due to the higher consanguinity rates in the region. According to Alfadhel et al. [35], the rates of consanguineous marriages are estimated to represent 51–56% of all marriages in Saudi Arabia and the regional variation of MMA shows a correlation with the tribal distribution across the country [35].

Throughout the years, a minor increase in MMA frequency was noticed in Western countries, possibly due to the introduction of newborn screening programs for inherited metabolic disorder [63] and/or increasing immigration rates in the developed regions [64]. In the region of MENA, in contrast, the decreasing tendency might be attributed to the changing cultural habits and norms and more conscious family planning, which are likely to reduce the frequency of consanguineous marriages [65, 66].

As in the vast majority of cases the reported data was not stratified by race/ethnicity, it was not possible to adjust for this factor in the conducted meta-analysis. However, it was addressed by three studies [31, 58, 61]. Lim et al. [58] found that the ethnic distribution of diagnosed cases was comparable to that of the national population in Singapore [58]. Feuchtbaum et al. [61] analyzed the racial/ethnic distribution of MMA subtypes in the United States and found that MMA mut^−^ is more frequent among the Middle Easterns, Filipinos, Vietnameses and Southeast Asians, while MMA mut^0^ seemed to be more frequent in Asian East Indian, Chinese and Filipino racial/ethnic groups [61]. Relatively high birth prevalence was reported in Southern Spain by Juan Fita et al. [31] which was explained by the growing immigration rates from North Africa and South America as 6 of the 10 positively screened children originated from parents arriving from the abovementioned regions [31]. These studies may indicate that investigating the ethnicity distribution of diagnosed cases may provide a more accurate picture on disease occurrence.

Due to the rarity of MMA, broadly targeted population-based prevalence studies are not available. However, the reports on the results of newborn screening programs provided valuable, high quality data on the birth prevalence of the disease. One limitation of the analysis is the lack of false positive and false negative data in newborn screening studies; however, where these data were available, the number of positive cases was adjusted. For many newborn screening studies, the follow-up time was not sufficient to appropriately assess the effect. In addition, many reports did not provide the rate of population coverage or the diagnostic tools and algorithms used to define the MMA subtype. To summarize, a newborn screening that includes limited gene sequencing and applies appropriate follow-up can be the “gold standard” for measuring prevalence of most metabolic disorders and possibly non-metabolic genetic disorders as well.

The terminology of the epidemiological measures used in the publications was inconsistent and heterogeneous. One strength of our study is the recategorization and harmonization of all published epidemiological measures.

The I^2^ statistics indicated substantial heterogeneity across the studies that underlines the necessity of random effects for the meta-analyses. The heterogeneity in the reported detection rates can be only partly explained by the differing type of reference population (screened newborns or all births) and calculation methods (birth prevalence, cumulative incidence in birth cohort, lifetime risk at birth) as the conducted sensitivity analysis (pooling together only birth prevalence data) indicated only minor differences compared to the base-case analysis (Table 1). Other confounders should be also taken into account. For example, the number of undiagnosed cases due to the different public health practices and economic status of the countries could influence the results.

Our findings align well with epidemiological studies published by Peng et al. [67] and Chapman et al. [62] after our review was performed [62, 67] and the systematic literature review by the Spanish Health Technology Assessment Agency**,** which was conducted with the purpose of evaluating the effectiveness of newborn screening programs [68]. However, compared to this work, our review was not restricted to screening programs, therefore, it provides a more comprehensive overview on disease epidemiology. Additionally, isolated MMA caused by mutase deficiency was investigated separately.

## Conclusion

In several countries, newborn screening programs provide relatively good estimates of the birth prevalence of MMA. However, a considerable evidence gap can be observed in certain geographical regions (e.g. South America, South Africa, Eastern Europe or Russia). The conducted systematic literature review and meta-analysis indicate a geographically uniform disease prevalence with the exception of MENA and confirms that MMA and its subtypes are ultra-rare disorders.

## Additional file

Additional file 1: **Table S1.** Search strategies and number of hits in different databases. **Table S2.** Exclusion criteria during the title and abstract screening. **Table S3.** Definitions of epidemiological measures. (DOCX 20 kb)

## Abbreviations

CINAHL: The Cumulative Index to Nursing and Allied Health Literature
CRD Database: Centre for Reviews and Dissemination
E-IMD: European registry and network for Intoxication type Metabolic Disorders
MENA: Middle East and North Africa
MMA mut: Methylmalonic acidemia/aciduria caused by the deficiency of methylmalonyl-CoA mutase
MMA mut^−^: MMA due to partial mutase deficiency
MMA mut^0^: MMA due to complete mutase deficiency
MMA: Methylmalonic acidemia/aciduria
OMIM: Online Mendelian Inheritance in Man
