# Supplementary material for: Systematic literature review and meta-analysis on the epidemiology of methylmalonic acidemia (MMA) with a focus on MMA caused by methylmalonyl-CoA mutase (mut) deficiency
Source: Orphanet J Rare Dis. 2019 Apr 25;14:84. doi: 10.1186/s13023-019-1063-z (PMC6485056; doi:10.1186/s13023-019-1063-z)
Supplement: Supplementary file 1 — Table S1. Search strategies and number of hits in different databases. Table S2. Exclusion criteria during the title and abstract screening. Table S3. Definitions of epidemiological measures. (DOCX 20 kb) [file 13023_2019_1063_MOESM1_ESM.docx]

additional file 1

Table S1 Search strategies and number of hits in different databases

| Search No. | Search area | Search | Date of search | Number of hits |
| --- | --- | --- | --- | --- |
| SCOPUS  (Medline, Embase) | | | | |
| #1 | Disease | ( ( ( TITLE-ABS-KEY ( methylmalon* AND ( acidemia OR acidaemia OR aciduria ) ) OR TITLE-ABS-KEY ( methylmalonyl-coenzyme AND mutase ) OR TITLE-ABS-KEY ( methylmalonyl-coa AND mutase ) OR TITLE-ABS-KEY ( "organic acidemia" OR "organic acidaemia" OR methylmalonicaciduria OR methylmalonicacidemia OR methylmalonicacidaemia ) ) ) OR ( TITLE-ABS-KEY ( mutase AND ( "mut0" OR "mut(0)" OR "mut-" OR "mut(-)" ) ) ) ) | 02/10/2017 | 2,650 |
| #2 | Clinical burden | TITLE-ABS-KEY ( inciden* OR prevalen*  OR demograph* OR epidemiolog* OR frequen* OR rate OR distribut* OR "lifetime risk" OR survival OR mortality OR outcome OR "natural history" ) | 02/10/2017 | 17,264,869 |
| #3 | Combined search - Clinical burden | #1 AND #2 | 02/10/2017 | **834*** |
| EBSCO  (CINAHL) | | | | |
| #1 | Disease | ( ( methylmalon* AND (acidemia OR acidaemia OR aciduria) ) ) OR ( methylmalonyl-coenzyme AND mutase ) OR ( methylmalonyl-coa AND mutase ) OR ( "organic acidemia" OR "organic acidaemia" OR methylmalonicaciduria OR methylmalonicacidemia OR methylmalonicacidaemia ) OR ( mutase AND ("mut0" OR "mut(0)" OR "mut-" OR "mut(-)" )) | 02/10/2017 | 48 |
| #2 | Clinical burden | inciden* OR prevalen* OR demograph* OR epidemiolog* OR frequen* OR rate OR distribut* OR "lifetime risk" OR survival OR mortality OR outcome OR "natural history" | 02/10/2017 | 974,052 |
| #3 | Combined search - Clinical burden | #1 AND #2 | 02/10/2017 | **21*** |
| EBSCO  (Academic Search Complete) | | | | |
| #1 | Disease | ( ( methylmalon* AND (acidemia OR acidaemia OR aciduria) ) ) OR ( methylmalonyl-coenzyme AND mutase ) OR ( methylmalonyl-coa AND mutase ) OR ( "organic acidemia" OR "organic acidaemia" OR methylmalonicaciduria OR methylmalonicacidemia OR methylmalonicacidaemia ) OR ( mutase AND ("mut0" OR "mut(0)" OR "mut-" OR "mut(-)" )) | 02/10/2017 | 648 |
| #2 | Clinical burden | inciden* OR prevalen* OR demograph* OR epidemiolog* OR frequen* OR rate OR distribut* OR "lifetime risk" OR survival OR mortality OR outcome OR "natural history" | 02/10/2017 | 5,337,890 |
| #3 | Combined search - Clinical burden | #1 AND #2 | 02/10/2017 | **205*** |
| CRD |  | methylmalon* OR "organic acidemia" OR "organic acidaemia" OR mut | 02/10/2017 | **7*** |
| PROSPERO |  | methylmalon* OR "organic acidemia" OR "organic acidaemia" OR mut | 02/10/2017 | **4*** |
| COCHRANE REVIEWS | Disease | (methylmalon* and (acidemia or acidaemia or aciduria)) or (methylmalonyl-coenzyme and mutase) or (methylmalonyl-coa and mutase) or ("organic acidemia" or "organic acidaemia" or methylmalonicaciduria or methylmalonicacidemia or methylmalonicacidaemia) or (mutase and ("mut0" or "mut(0)" or "mut-" or "mut(-)")) in Title, Abstract, Keywords | 02/10/2017 | **11*** |
| Rare disease organizations | Orphanet | Free search with relevant disease terms | 02/10/2017 | **3*** |
|  | EIMD |  |  | **1*** |
|  | NORD |  |  | **10*** |
|  | CORD |  |  | **0*** |
|  | EURORDIS |  |  | **0*** |
|  | NIDDK |  |  | **9*** |
|  | GARD |  |  | **0*** |
|  | CLIMB |  |  | **0*** |
|  | TALK |  |  | **0*** |
|  | OAUK |  |  | **2*** |
|  | OAA |  |  | **8*** |

*Hit number of final search strategy

Table S2 Exclusion criteria during the title and abstract screening

| Irrelevant title without English abstract | All articles with irrelevant titles and without English abstract were excluded at this step. |
| --- | --- |
| Not related to MMA | Since the literature databases cover a wide variety of scientific areas, studies that have no relevance to MMA were excluded at this step. |
| Editorial, letter, conference abstract or review | Editorials, letters, conference abstracts and non-systematic reviews were excluded at this criterion. All systematic literature reviews that seemed to be relevant for the epidemiology of MMA were included. |
| Case study or case series | Due to the rarity of the disease a minimum patient number was not defined, but if the abstract defined the paper as a case study or a description of a few case studies (the study did not publish any estimation of disease occurrence or aggregated results), then the study was excluded at this step. |
| No relevant data on epidemiology/natural history of MMA | All studies that contained no relevant data on the epidemiology/natural history of MMA were excluded at this last step. |

Table S3 Definitions of epidemiological measures

| Measure | Definition |
| --- | --- |
| Birth prevalence | Number of cases with a birth defect in a defined area and time period divided by the number of live births in that area and time period. National aggregate statistics and newborn screening programs with a high sensitivity and specificity and ~100% population coverage are considered to be the most reliable sources to measure the birth prevalence in a population. |
| Estimated birth prevalence | The identified (and subsequently confirmed) cases during a period of newborn screening are divided by the number of screened newborns in the same period of time. A newborn screening program without ~100% coverage can only provide estimations on the true birth prevalence in the population. |
| Cumulative incidence in the birth cohort | The newly diagnosed cases within a specified birth cohort during a period of time are divided by the total number of subjects in the birth cohort. The observational period is equal to the period of birth cohort. |
| Lifetime risk at birth estimated by the diagnosis period method | The newly diagnosed cases in a period of time are divided by all live births in the same period of time. Lifetime risk is a special case of cumulative incidence in which the period of time studied is the entire remaining lifetime. The diagnosis (DX) method is calculated as the number of patients with a particular (or specific) diagnosis in the observational period divided by the number of live births during the same period^[[1]](#footnote-1)^. |

1. Foss AH, Duffner PK, Carter RL: Lifetime risk estimators in epidemiological studies of Krabbe disease: review and Monte Carlo comparison. *Rare Diseases* 2013, 1:e25212. [↑](#footnote-ref-1)
